# Transactions of the Medical Association of Pennsylvania, 1884

**Published:** 1885-02

**Authors:** 


### Book Reviews.

---

TRANSACTIONS OF THE MEDICAL SOCIETY OF THE STATE OF PENNSYLVANIA at its thirty-fifth annual session, held at Philadelphia May 14, 15, 16, 1884.

This book of 622 pages, including reports of county societies, with list of members, is a worthy representation of the progress of the medical profession in that great centre of instruction where this annual meeting occurred, and appeals to the profession in the United States for imitation, not only in the character of the papers but in the order and style of the work presented to the public.

The example of this society in having a salaried, permanent Secretary, calls for the attention of other similar organizations, as the great amount of labor which devolves upon this officer in the proper discharge of so onerous a duty in behalf of a common and general interest of the members, should not be imposed upon any individual without compensation for this important service. Though competent men are found to undertake it and perform it faithfully without pay, the honor of such position, and the grateful appreciation by others of the proper execution of the task, does not relieve such a body of the responsibility for mitigating the hardships of preparing an elaborate report of their proceedings by a pecuniary recognition of the labor.

The complete and thorough organization of the State and county societies, with their co-operation in the advancement of practical medicine, is a striking feature of this report of their transactions; and the large representations from localities outside of Philadelphia which attended these meetings, shows the interest taken by the members of the local societies.

The State society imposes no assessment on the county societies, or the individual members thereof, other than that for the Transactions. Among the regular members some half dozen names of females appear on the record, and outside of these, eight are made members by invitation.

An interesting phase of the Transactions is the report of two meetings of the American Medical Association, this body having met twice in the interval of the annual reunion of this Society; and Dr. J. H. Packard requested, in referring to the action as to the Journal of the American Medical Association, that the report be amended, to simply say that the minority report was defeated, which was so corrected.

The Society indorsed the action of the American Medical Association, setting forth "that experimentation on animals is a most useful source of knowledge in medical sciences."

Notwithstanding the objection of Dr. H. C. Wood, that it was not the place of this Society to prepare a plan for organizing another society, it was "Resolved, That a committee of seven be appointed to prepare and report at this session a plan for organizing a society for securing a State Board of Health." The chairman of this committee, Dr. E. A. Wood, subsequently made a report recommending "to the people the organization of a State Society for the purpose of encouraging the establishment of a State Board of Health."

The Recording Secretary, Dr. H. Leffman, reported to the Society that the Chairman of the Board of Examiners, appointed by the Philadelphia County Society, stated that no candidates were referred to the said Board for examination during the year.

The large proportion of very prominent members of the profession in its various departments, which enters into the composition of this Society, gives it an importance and weight in the medical scales that no similar organization can claim in this country. Since the disruption of the New York State Association and the division of the members of the medical profession into the two Societies, there no longer exists a competitor for this palm of distinction as the champion Medical Society of North America, and the Pennsylvania State Medical Society is *facile princeps* among similar organizations in these United States.

Not alone in the nature of the material which composes it, but by its numerical strength, no other similar organization can wield so much power, and there are 2,012 members of the county societies represented in this body of 503 registered on the list. The surplus fund, after meeting all expenses of the last annual meeting, is $2,451.65, giving a status financially which is unequaled, and only $79.50 of dues unpaid by county societies.

It is somewhat remarkable that with the means in hand to encourage original investigations, no premium for prize essays is offered by this Society. Whether it may be on principle that such stimulus for individual effort is withheld is not apparent, but all know that there are many of the younger members of the medical profession to whom the prominence given by awarding a premium for a special work of this kind is desirable, even should the pecuniary consideration be of no moment.

It would seem that there was a general want of the initiative element among the members of the Society, as out of some thirty papers included in this volume very few of them indicate any profound research of a scientific nature,and still less of the original initiative element in the experimental department. In view of the practical developments from clinical observations reported in many of the contributions the general practitioner should rest satisfied.

In connection with a eulogy upon Dr. S. D. Gross, we find the following: "Be it therefore resolved, that we, the members of the Medical Society of the State of Pennsylvania, now in session in Philadelphia, express our deep grief at the death of the ex President of our Society, and our heartfelt sympathy and condolence with the members of his bereaved family. Resolved, th t we do most heartily approve of the resolution passed by the Alumni Association of the Jefferson Medical College, namely, that an enduring monument in marble or bronze be erected in Fairmount Park to commemora e his greatness for generations to come."

It impresses the reader of the programme very favorably to find the subjects accompanied by an abstract of the views presented, and if we were inclined to pass over the different papers with a cursory notice, these succinct points might be given, but the great importance of many papers contained in this volume of Transactions demands a more detailed view of their practical bearings.

The scholarly address of Dr. H. H. Smith, President of the Society, covers the past history of medical organization for the promotion of the various branches which are essential to the physician and surgeon. It is a comprehensive resume of medical advancement during the present century, under the guidance of good and true men, who have devoted themselves to the building up of the temple of science; and with all the drawbacks from quackery in different forms, presents the fair proportions of a growing structure, which must eventually, like the noble monument which graces the grounds of our national capital, receive the capstone of final triumph, through the energy and constancy of the master-builders in our profession. The matured reflections which are so cogently arranged in this masterpiece of professional analysis, deserve a consideration from the members of all similar associations, which can only be extended to them by a careful perusal of the address, and it is to be hoped that it may be republished and extensively distributed.

In an address on "*the present outcome of sanitary agitation*," the vexed question of hygiene is summarily disposed of by Dr. Benjamin Lee. He claims that legislation is not destined to accomplish satisfactory results and that it must be undertaken by the organization of self-protection associations in each community. A model for a charter and by laws for "Articles of Association of the Citizens' Protective Sanitary Association" is presented; but the great difficulty is not in impressing people with the great advantages of hygienic regulations so much as in getting any efficient co operation for any definite result.

The indorsement given in the paper for certain business corporations, which fill the hiatus existing in our large cities between the appreciation of the evil and the application of the remedy, savors rather much of an advertisement for these same stock companies. Yet if this thing will not be done by public functionaries nor by the interested parties, they should not play the dog in the manger.

The address on "Mental Disorders," by Alice Bennett, M. D, presenting the "relations of heart disease to insanity," may be relegated to its proper field for the consideration of alienists.

Dr. W. H. Daly commences his address in medicine with some thrusts at the germ theory and Listerism, stating afterwards that the richest of all the great fields in our healing art is that "in which every man is competent to do lasting, useful work by careful clinical observations carefully recorded and carefully reported." We recognize this point as well taken for practitioners, and would urge the importance of the case book, in which the diagnosis of all diseases treated should be faithfully set down, with their treatment and results, whether favorable or not. He advises that homœopathy be treated with silent contempt, and facetiously suggests that "if the doctrine and dosage of the dogmatist are palatable, and their patients like them, why, in God's name, let them take them, and take them until, at length, they find there is nothing in them." And we say, so mote it be.

As the first practical point of the address in obstetrics Dr. Jacob Price reiterates his treatment for "pernicious vomiting of pregnancy," which, it will be remembered, was formerly urged by him in the application to the os uteri of a solution of iodine, crystalized carbolic acid and tannin, each two drachms, dissolved by the aid of heat in an ounce of pure glycerin. In some cases he adds morphine and atropia. A pledget of absorbent cotton saturated with pure glycerin, followed by a dry one, is placed in contact with the os uteri afterwards. This is repeated at intervals of three days, and "usually not more than three or four of these applications are required" to effect a cure.

In explanation of this course he says: "Step by step I reached the conclusion that in these unmanageable cases of vomiting during pregnancy, there was present congestion or inflammation of the uterine cervix. In no cases did examination fail to disclose the presence of local diseases of this character, and treatment based upon this view proved eminently satisfactory." Cases are reported of its success, and the names of colleagues given in corroboration of its salutary effect.

If the appeal for a board of health for Pennsylvania, by E. A. Wood, M. D., had been put in a form to be comprehended by the people, instead of coming with such a flourish of trumpet over the would-be discovery of the comma bacillus by Koch, it might have led at least to the removal of filth from their premises. But it has unfortunately become fashionable to chase germs, like a jack-o'-the-lantern, over bogs and quagmires, until few can be found to practice common sense for the eradication of impurities in the atmosphere by attention to cleanliness.

The prime difficulty with the boards of health thus far has been the lack of proper support by the people and the remissness of individuals in domestic hygiene, owing largely to the impractical and impracticable presentation of this matter by those who urge attention to public health by setting "the scientist at work with his methods and implements," instead of plunging into the cesspools and delving into the masses of corruption which poison the atmosphere of their homes.

Proper medical education is held by Dr. Henry Leffman to be that which fits students for a specialty in practice; whereas those who regard the training in medical colleges as a preparation for any and all classes of general practice would eschew completely the setting up any preconceived line of distinction in the course of studies. The fundamental principles of pathology, physiology and therapeutics being founded on a thorough understanding of the structure of the physical frame, it is incumbent upon those who may select some special field of duty to have this elementary knowledge, and we fail entirely to see the force of the illustration from dentistry in favor of original distinctions in the rudimentary studies for medical or surgical specialties. The post-graduate schools are the proper field for the development of specialties, and while many who expect to engage in general practice may profit after receiving the title of M. D., yet the polyclinic is emphatically the school for the full training of all specialists.

After reading the address in ophthalmology, by W. S. Little, M. D., touching the value of pupillary symptoms in general disease, the full scope of an analysis of one thousand symptoms fails to be comprehended; and no doubt the author may fall back upon the self congratulatory solution that he cannot furnish ideas with brains likewise to his reader. But we are forcibly reminded at the conclusion of this address of the remarks of a colored preacher, that in the outset he would tell his hearers something that he knew more about than they did; secondly, he would treat of something they were better acquainted with than he was; and, lastly, he would speak of matters which neither understood, and about which neither he nor they knew anything definitely. While this concession is not made on the part of the author for himself, he must excuse us for the inference that he has made all as clear as mud to those who are not able to see as he sees the pupillary changes on page 165.

The dogmatic attitude assumed by Dr. John B. Roberts, when making his address on Surgery, warrants us in saying that the scarecrow he presents in regard to the inhalation of chloroform is not qualified as it should be by reference to the precautions in its administration which reduces the risk, so that surgeons are justifiable in resorting to this prompt and efficient anæsthetic in most operations.

His off-hand shot at styptics is wide of the mark, as they often serve under circumstances which preclude other measures for arresting hemorrhage, which has been fully verified by those having the largest experience in rectal and urethral surgery and by all in alveolar hemorrhage, epistaxis, etc.

The injunction to incise before pus has yet been formed in acute phlegmonous inflammation, rather than wait for such thinning of the overlying tissues as will make the pus apparent, is no more warrantable than the antiquated crucial incisions in cases of carbuncle, which are, or should be, abandoned for other means that are more efficient and less painful to the patient.

While treating of "hygiene in the public schools," Dr. Thomas H. Fenton remarks that ventilation is perhaps the most important in school hygiene. With close, foul rooms, in which the air, poisoned by the exhalations of the bodies, is respired several times by occupants, incalculable harm is produced. "Carbonic acid, when in considerable amount, is believed to produce evil effects upon the system, but the languor and oppression, the headache and flushing which result from deficient ventilation are consequent rather upon the deficiency of oxygen in the air together with its organic foulness."

Dr. George Ross states that each person requires a continuous supply of about sixteen cubic feet of air per minute, moving at the rate of 150 feet in that time and diffused through a space containing 320 cubic feet, the size of the opening for the admission of air being about two square inches.

The adaptation of seats to the size of the pupil is urged as important, and he adduced facts illustrating the disadvantages from improper illumination, either by the deficiency of light or its irregular distribution.

The protective rights of the insane are presented by R. H. Chase, M. D. With the proper observance of existing provisions, he says, "it is to be hoped, and can be confidently expected, that public distrust will give way to confidence in our public and private institutions." The general Board appoints a local committee of visitors for each county, and one or more members of the Lunacy Committee visit every asylum and place where the insane are confined twice, at least, every year. Stringent regulations are enforced to prevent abuse of the insane, and any one guilty of it shall be prosecuted according to law. The greatest liberty is allowed in communicating with friends by letter or otherwise, and letters addressed by a patient to his counsel or to the Lunacy Committee are required to be sent forthwith, unopened and without inspection, under severe penalty for violation.

With such regulations in force, it is not likely that any one could be detained unjustly in a lunatic asylum of Pennsylvania for many days.

Dr. Charles W. Dulles has evidently made extensive search after "disorders mistaken for hydrophobia," and most assuredly has proved that there are numerous forms of disease which so much simulate this affection as to have deceived the observers if the cases were not really of that class styled hydrophobia.

In a former publication he left the reader to infer that the existence of such a disease as this peculiar excitation of the nervous system was involved in doubt as a consequence of being bitten by a dog under the influence of rabies, and with the weight of testimony recently accumulated in the experiments of M. Pasteur, his views as to the reality of hydrophobia do not seem to have undergone any material modification.

He should consult the historic doubts in regard to the person and deeds of Napoleon Bonaparte before letting his skepticism carry him into greater excesses, as the most that his cases could prove is that other diseases simulate the *veritable* hydrophobia.

Dr. Arthur van Harlingen elucidated the principles of external treatment in diseases of the skin, by a methodic division of the various remedies into eight different classes: 1. Protectives; 2. Sedatives; 3. Astringents; 4. Anæesthetics; 5. Stimulants; 6. Caustics; 7. Mechanical measures; 8. Parasiticides.

Dr. John V. Shoemaker treats of jequirity in diseases of the skin, and quotes Dr. Arthur Benson's report before the Ophthalmological Society of the United Kingdom, stating that he had a high opinion of the treatment of granular lids with jequirity. Its use has been extended to other ulcerated and granular surfaces, and in cases where one application does not suffice, and where there is still evidence of unhealthy granulation, a second, third or fourth application may be made, as the case may require. For this purpose he devised a more effective preparation of greater strength and more viscid, so as to longer adhere to the surface, which presents all the appearance of an emulsion, and is applied with a camel's hair pencil or mop. If used on weak and irritable patients, it may give rise to great constitutional disturbance.

Dr. J. H. Musser has modified the base of the sphygmograph, and claims greater ease and rapidity of application and greater uniformity and clearness in the tracings for this instrument.

Dr. Joseph B. Potsdamer quotes Buckler and Senator in support of his position that bronchitis and pneumonia occur as the complications of rheumatism stating that pneumonia is always secondary to rheumatic bronchitis, and that proper rheumatic treatment will often save the patient.

A plea for chemistry by Trail Green, M. D., has a special point in cautioning all concerned against explosive compounds in prescriptions.

Dr. Benjamin Lee recommends massage—the latest handmaid of medicine—as a remedial agent of great value, and begs us to remember that he does not present its claims unadvisedly. In these days of walking matches and base ball this process ought to be in demand.

Diphtheria is regarded by L. B. Kline, M. D., as a specific and infectious disease of a parasitic nature, for which he seems never to have heard of the mercurial treatment.

With the most profound appreciation of the "work of women physicians in Asia," by Mary H. Stinson, M. D, let us make her a bow and pass on.

From the description of "obstetrical forceps, jointed at the junction of the blades and shanks," by J. A. McFerran, M. D., it is to be inferred that when the blades are once secured upon a child's head we have nothing more to do than haul away. "And even when traction is made from the extremity of the handles, the joint leaves the head free to deflect and pass an obstruction with the greatest possible ease."

The nonchalance that is manifested by Dr. Edward Jackson as to alarming and dangerous doses of the mydriatics, reminds us of the various applicants for a place as coachman, and the selection of the one who responded to the interrogation as to how close to an immense precipice he could drive, that he would keep as far off as possible. Moral—Run no risks in giving poisonous medicines.

The idiosyncrasies of individuals in regard to the effects of this class of drugs call for special attention.

The disquisition touching "a form of epithelial mycosis," by Albert G. Heyl, M D, has, amongst other well elaborated points, the following phrases: "It cannot be doubted that we must look upon the skin not simply as a protective vestment for the body, but as an organ intimately associated with the internal organs." He further states that "Cohnheim has traced the nerve filaments into the epithelial layers."

"Heitzman says that he has observed the nerve filament in actual connection with the epithelial cell. This would seem to be clinically confirmed by the disease under consideration. If this be so, the epithelial cell is something more than a protection element for the underlying tissues; it is the terminal element of a centripetal nerve filament." Here is the whcle solution in a nutshell.

The "electric laryngoscope," by Carl Seiler, M. D., claims to be an improvement upon all previous modes of looking into dark holes, and it certainly combines the qualities of affording unobstructed vision with a highly developed illumination.

The question by Charles S. Turnbull, M. D., "does a chronic discharge from the ear make life insurance hazardous?" may be rid of some of its embarrassments by using the following within the cavity:

℞—Creosote, 20 drops; acetic acid, 60 drops; distilled water, 10 ounces. Mix, and pour a little into the ear night and morning, allowing it to run out after one minute.

Ophthalmological observations in Will's eye hospital, by Peter D. Keyser, M. D., have a practical bearing in the drawing of an improved cystotome; but we might have been spared the sight of those diseased protruded eyeballs at the close, from a photograph taken the next day after death.

A contribution to the operative treatment of purulent pleural effusions, by Edward T. Bruen, M. D., and J. Wm. White, M. D., is a fair resume of the recognized practice in such cases, even when most complicated. It is worthy of the careful consideration of those who may need light and guidance in connection with empyema and the procedure for its relief.

The recommendation for bichloride of mercury as a surgical dressing by C. B. Nancrede, M. D., is based upon the supposition that a germicide is required in these cases, and from the experience we have had with it in the treatment of itch, a high place ought to be awarded to it in this point of view. But the question comes home to all as to the absorption of this poison and the liability to cause more harm to the general system than the said parasites. The caution which experience has sanctioned in regard to carbolic acid, urges itself more forcibly still respecting the app ication of solutions of corrosive sublimate to raw surfaces.

The inquiry by Dr. DeForest Willard "is excision of the tarsus necessary in the club-foot of children?" places the whole treatment of such cases upon the basis of gradual and forcible correction, which most assuredly should be persisted in while there is the most remote prospect of benefit, rather than resort to unnecessary resection of the bony structure. Time and patience are important elements of success in treating club-foot.

It must strike every reader as a strange omission in the report of Dr. W. S. Janney's case of excision of trachea, that up to the date of the meeting of the society, or when the Transactions went to press, no further notice was given of the final result under such critical surroundings.

The presentation of two splints by Dr. Benjamin Lee, giving a clinical illustration of the value of combining motion with extension in the treatment of diseases of the hip joint, and of fixidity, speaks louder than words in favor of mobility so as to prevent anchylosis. A report made by Dr. DeF. Willard, upon request of Dr. Lee for a committee to examine his patient, indicates a most satisfactory result of his treatment.

Outside of the general proceedings of the Society, and the various contributions noticed, there are additional reports from the County

Societies included in this volume, comprising much valuable matter, of which our space does not allow any notice, but will repay careful perusal. J. McF. G.

